# The Effect of Sleeve Gastrectomy on Oxidative Stress in Obesity

**DOI:** 10.3390/biomedicines8060168

**Published:** 2020-06-19

**Authors:** Alessio Metere, Claire E. Graves, Donatella Pietraforte, Giovanni Casella

**Affiliations:** 1 Surgical Sciences Department, “Sapienza” University of Rome, Viale Regina Elena 261, 00161 Roma, Italy; giovanni.casella@uniroma1.it; 2 Department of Surgery, University of California, San Francisco, 1600 Divisadero St. 4th Floor, San Francisco, CA 94115, USA; claire.graves@ucsf.edu; 3 Core Facilities, Istituto Superiore di Sanità, Viale Regina Elena 299, 00161 Roma, Italy; donatella.pietraforte@iss.it

**Keywords:** sleeve gastrectomy, oxidative stress, electron paramagnetic resonance (EPR), bariatric surgery

## Abstract

High concentrations of free radicals are present in the blood of obese patients. Free radicals are associated with endothelial dysfunction, diabetes, and neoplastic transformation, all conditions that are closely related to obesity. The purpose of our study was to determine whether bariatric surgery modifies the production of free radicals in obese patients. In total, 20 patients with morbid obesity, who were candidates for laparoscopic sleeve gastrectomy (SG), and 18 controls were enrolled in the study. Oxidative stress was studied in obese subjects before and after sleeve gastrectomy. The evaluation of oxidative stress was carried out on blood samples using electron paramagnetic resonance, a refined spectroscopic technique used to identify and quantify the major free radicals, such as ^•^OH, O_2_^•^, ONOO^-^, and NO. Oxidative stress was higher in subjects with morbid obesity prior to surgery, compared to the controls (CP• 9.9 ± 0.3 µM vs. 5.8 ± 0.2 µM). After SG, values decreased to levels comparable to those of controls (CP• 5.4 ± 0.2 µM). Further analysis identified O_2_^•^ as the main free radical responsible for the oxidative stress. Obesity is associated with an increased blood concentration of free radicals. The normalization of free radicals after sleeve gastrectomy highlights another important benefit of this bariatric surgery technique.

## 1. Introduction

The prevalence of obesity continues to rise in Western countries. With obesity comes obesity-related conditions, including cardiovascular disease, stroke, and type 2 diabetes [1]. Moreover, multiple cancers are associated with obesity [2,3], including thyroid [4], kidney [5], breast [6], pancreatic [7], prostate [8], and endometrial cancers [9]. Though the associations between obesity and these diseases have been well studied, less is known about the pathological pathways that connect them. One possible factor may lie in the presence of increased markers of oxidative stress, associated with lower antioxidant enzymes, in obese patients [10]. Reactive oxidizing species (ROS) are associated with systemic inflammation, endothelial dysfunction, and atherosclerosis, all factors related to cardiovascular diseases [11,12]. Moreover, oxidative stress plays a significant role in cancer, due to the chemical modification induced by an excess of free radicals that interfere with the cellular signaling responsible for cellular proliferation [13,14,15,16].

**Figure 1:**
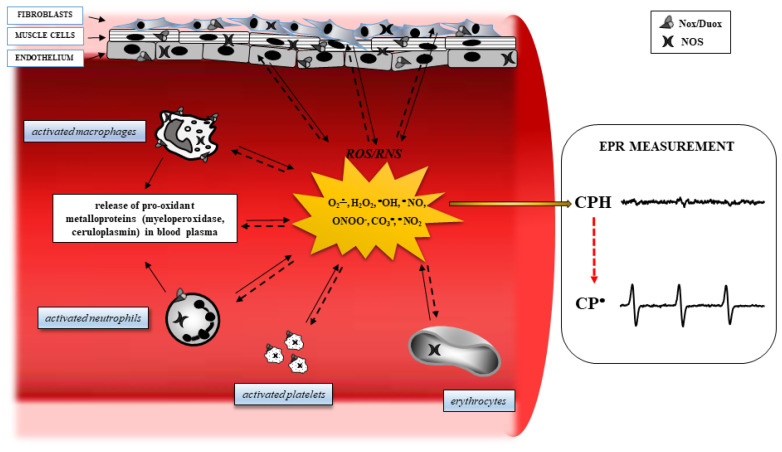
Oxidative stress pathways in a blood vessel. In several degenerative diseases, diabetes, and cancer, inflamed tissues and blood cells release Reactive Oxygen Species (ROS) derived from oxygen and nitrogen, following the activation of specific intracellular enzyme systems (continuous arrows), such as NADPH oxidase (Nox), dual oxidase (Duox), and nitric oxide synthase (NOS). The ROS formed can oxidize important targets in tissues, including proteins, enzymes, nucleic acids, lipids, carbohydrates, and antioxidants (dashed arrows), leading to alteration of intracellular signaling and cell functions, and boosting cells to death. Electron Paramagnetic Resonance (EPR) spectroscopy reveals ROS formation by monitoring the oxidation of the spin probe 1-hydroxy-3-carboxy-pyrrolidine (CPH). This compound does not show an EPR-detectable signal, but it is rapidly oxidized to the related EPR-detectable CP• in the presence of ROS.

Obesity is strongly associated with the condition of chronic inflammation [17] and an increased production of ROS. For example, obese patients show an overactivation of inducible nitric oxide synthase (iNOS), resulting in nitric oxide (NO) overproduction, which is related to insulin resistance [18]. Evidence has demonstrated that when expressed iNOS is fully active, it can generate a large amount of NO to react with O_2_^•^, resulting in elevated concentrations of peroxynitrite (ONOO^−^), a powerful reactive oxidant [19,20]. In addition, hyperglycemia, frequently present in obese patients, increases the levels of inflammatory cytokines, such as IL-6, TNF-a, and MCP-1, which are then able to increase the production of ROS [21]. In obesity, the pro- and anti-inflammatory effects are mediated by molecules, such as NF-kappaB, AMPK, JNK, PKC, PKA, and IKK-beta, can be modulated by oxidative stress, giving rise to the chronic inflammation frequently present in obese patients [22,23]. Furthermore, an excess of ROS is capable of damaging cellular macromolecules, modifying the structure of lipids, proteins, and DNA, and resulting in a decrease in protein synthesis, alteration of normal cellular physiology, and decreased resistance to environmental stresses (Figure 1).

As previously described, several molecules and biomarkers have been implicated as potential mediators in the link between obesity and chronic disease that could play a key role in the development of obesity-related diseases. Furthermore, these molecules could potentially provide an opportunity to identify patients at high risk for these diseases before they develop. These “obesity biomarkers” include hormones secreted by adipose tissue, such as adipokines, or circulating hormones, such as cytokines, as well as markers of oxidative stress. Because of the wide-reaching effects of oxidative stress, and its implication in multiple obesity-related conditions, such as cancer and cardiovascular and respiratory disease, we focused our attention on biomarkers of oxidative stress. In recent years, bariatric surgery, such as sleeve gastrectomy (SG), has proved particularly effective in reversing the negative health effects associated with morbid obesity [24,25]. Laparoscopic SG has demonstrated significant improvements in all of the risk factors associated with obesity, resulting in a decrease in mortality and morbidity, and a remarkable improvement in quality of life [26]. The purpose of our pilot study was to investigate the role of ROS in morbid obesity and the impact of SG on oxidative stress. Our aim was to directly measure and identify the free radicals involved in obesity using electron paramagnetic resonance (EPR) spectroscopy, and to evaluate whether SG results in decreased oxidative stress.

## 2. Experimental Section

### 2.1. Study Population

The study population was recruited at the Department of Surgical Sciences of the Umberto I Hospital of Rome and included 20 subjects (4 male and 16 female), aged between 27 and 55 years, with BMIs ranging 36.3–46.7 kg/m^2^ and eligible for SG according to the diagnostic, therapeutic, and healthcare management protocols of Italian Society of Surgical Obesity (SICOB) [27]. The control population was represented by 18 healthy donors (HD), age and sex matched, with a normal BMI 19.2–24.7 kg/m^2^. All procedures performed in the study involving human participants were in accordance with the ethical standards of our institutional research committee and the 1964 Helsinki declaration and its later amendments. Informed consent was obtained from all individual participants included in the study (approved by Umberto I Hospital ethics committee, code: 4180, 7 July 2016). Exclusion criteria were the presence of debilitating disease or the lack of informed consent and/or authorization form for the processing of personal data. Variables, such as age, gender, BMI before and after surgery, length of hospital stay, complications, weight loss, and remission of comorbidities, were recorded at the time of the recruitment and at 1, 3, 6, and 12 months. Blood pressure, fasting blood glucose (FBG), hemoglobin A1c (HbA1c), and serum lipids were checked at each visit. All subjects underwent electrocardiogram, chest X-rays, esophagogastroduodenoscopy, spirometry, routine blood exams deemed necessary for surgery, and psychological evaluation prior to SG. The blood samples for the measurement of biomarkers of oxidative stress were taken using heparinized tubes, to prevent any changes in the redox state of the blood, and completed within 2 h from collection. In the postoperative period, all patients were fed a liquid diet in the first month, followed by a soft diet until the third month. After three months, the patients were allowed a normal diet. Vitamin supplements were not prescribed routinely for patients with SG; they were recommended individually according to postoperative follow-up results.

### 2.2. Laparoscopic Sleeve Gastrectomy (SG)

All patients received antibiotic and thromboembolic prophylaxis and were operated in the semi-lithotomy position. The standard 4-port technique was used for SG. The first port was placed in the abdomen supra-umbilically and CO_2_ insufflation was established at a pressure of 12 mmHg. A 5-mm port was placed for liver retraction from the subxiphoid area. The working ports were placed under direct vision from the right and left subcostal lines. Gastric vessels along the greater curve were mobilized by using a vascular sealing device (Ligasure; Maryland, Covidien, CO, USA). The stomach was divided starting 2 cm from the pylorus until 1 cm to the angle of His vertically over a 36 Fr calibration tube by using 4 to 6 60-mm staplers (Covidien; Endo-GIATM, Tri-StapleTM, USA). In all patients, the staple line was over sewn with a running suture (V-Loc; Covidien, USA) to prevent bleeding, and a silicone drain was placed along the suture line.

### 2.3. Chemicals and Instruments

Spin probe 1-hydroxy-3-carboxy-pyrrolidine (CPH), 3-carboxy-proxyl radical (CP•), and Phorbol 12-Myristate 13-Acetate (PMA) were purchased from ENZO Biochem (Laufelfingen, Switzerland). Superoxide dismutase (SOD), diethylenetriaminepentaacetic acid (DTPA), H_2_O_2_, and catalase (Cat) were purchased from Sigma (St. Louis, MO, USA).

### 2.4. Preparation and Treatments of Whole Blood

Fresh heparinized human blood was transferred in Falcon 15 mL and balanced for 10 min at room temperature with air on a shaker to allow comparable oxygen partial pressure in the blood samples (pO_2_ = 207 ± 9 mmHg). To confirm the functionality of our experimental system and to demonstrate that the blood can be a suitable medium for the study of oxidative stress, we added 1 μM PMA to the blood samples with or without the presence of SOD, DTPA, H_2_O_2_, and Cat. PMA is able to stimulate white blood cells (WBC) to produce O_2_^•^ that consequently oxidizes CPH to generate CP•. Moreover, to identify the oxidant species, we pre-incubated 100 μL of whole blood with suitable scavengers/inhibitors (such as SOD, DTPA, H_2_O_2_, and Cat), for 15 min at 37 °C, then tested the modification of the CP• values, before and after the addition.

### 2.5. Analysis of Oxidative Stress Biomarkers by the EPR Technique

EPR spectroscopy reveals ROS formation by monitoring the oxidation of the spin probe, the cyclic hydroxylamine 1-hydroxy-3-carboxy-pyrrolidine (CPH). This compound does not show an EPR-detectable signal, but it is rapidly oxidized to the related EPR-detectable CP• in the presence of ROS. CPH was dissolved in PBS, pH 7.4, extensively treated with Chelex 100 to avoid any metal-catalyzed probe oxidation. CPH (1 mM) was added to 100-µL samples and, after 10 min at 37 °C, samples were drawn up into a gas-permeable Teflon tube with 0.81 mm internal diameter and 0.05 mm wall thickness (Zeuss Industrial Products, Raritan, NJ). The Teflon tube was folded four times, inserted into a quartz tube, and fixed to the EPR cavity (4108 TMH). EPR spectra were measured at 37 °C on a Bruker ECS 106 spectrometer (Bruker, Rheinstetten, Germany) equipped with a variable-temperature unit (ER4111VT). The gas flow was air. CPH oxidation was monitored by the formation of the characteristic 3-line spectrum with a hyperfine coupling constant of 1.63 ± 0.04 mT, attributable to the corresponding nitroxide radical CP•. The low field shoulder of this spectrum was chosen to quantify the CP•, because the middle component centered at g 2.0 overlaps with many other free radical signals found in biological systems. The intensity of the signal produced by the formation of CP• was calculated 10 min after the addition of CPH to obtain a good concentration of CP•. The spectrometer conditions common to all the spectra were the following: Modulation frequency, 100 kHz; microwave frequency, 9.4 GHz; microwave power, 20 mW; 1 × 104 gain, modulation amplitude, 0.1 mT; conversion time, 20.5 ms; time constant, 82 ms; sweep time, 21 s; number of scans, 1.

### 2.6. Statistical Analysis

All data were expressed as the mean ± SD of at least 3 measurements and analyzed using the Student’s t test and correlation test with the statistical software Graph Pad Prism 5.0. Values of *p* < 0.05 were considered statistically significant.

## 3. Results

### 3.1. SG Outcomes

At presentation, 5 (25%) patients had one or more comorbid condition: 3 (15%) patients with mild/moderate obstructive sleep apnea (OSA) and 4 (20%) patients with hypertension, on anti-hypertensive treatments (AHT) [28]. All patients ambulated freely on the first postoperative day and drank clear fluids within 24 h of surgery. The mean body weight before surgery was 114.3 ± 14.8 (range 91–144) kg and 67.6 ± 8.9 (range 52–85) kg one year after SG (*p* < 0.01). The mean BMI detected preoperatively was 42.3 ± 3.1 (range 36.3–46.7) kg/m^2^, which decreased progressively to 25.0 ± 2.2 (range 20.3–29.0) kg/m^2^ at one year after surgery (*p* < 0.01). Complete remission of OSA was achieved in all patients affected, by one year after surgery. A significant improvement in the control of hypertension was obtained in three of four patients, who no longer required AHT one year after SG (Table 1). No changes in BMI or other relevant clinical data were detected in the control group HD during the follow-up period.

### 3.2. EPR-Spin Probing Analysis of Oxidative Stress in Blood

**Figure 2:**
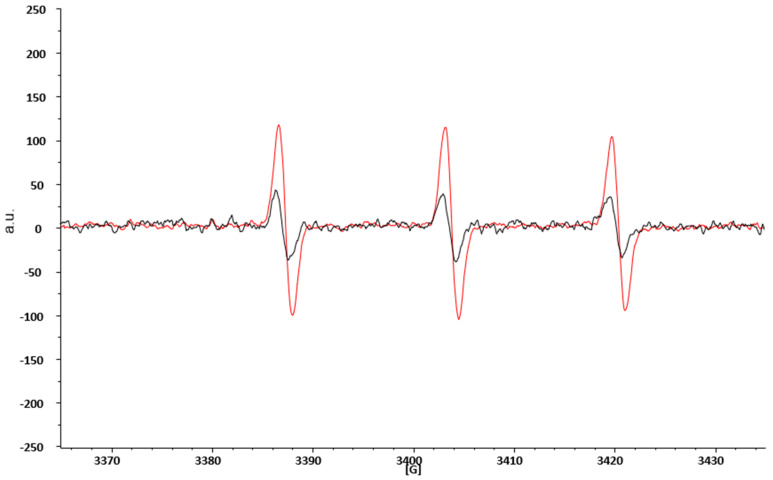
EPR spectroscopic spectra of CP• obtained from blood analyses of HD and obese patients prior to SG. a.u. = arbitrary units; G = gauss, the measure of the external magnetic field.

ROS are highly reactive species with a half-life usually less than 1 ms and hardly detectable by the direct EPR technique alone. This limitation is overcome by combining the EPR technique with spin probing. In brief, it consists of adding to the study system an organic compound (in our case the CPH), capable of reacting with the ROS present. The reaction product is a nitroxide radical (CP•), much more stable, and therefore with a longer lifetime, than the first radical, and easily detectable by the EPR technique [29]. Figure 2 represents two typical spectra obtained by EPR spectroscopic blood analyses. The black spectra are obtained from the blood of HD, while the red one are from obese patients before SG. We overlapped the spectra to show that in both cases, we detected the same signal (the shape of the spectra is the same), which represents the CP• formation. However, the intensity, measured as arbitrary units (a.u.) of the red spectra is increased in obese patients with respect to HD, thereby demonstrating that obese patients have an increased production of ROS.

**Figure 3:**
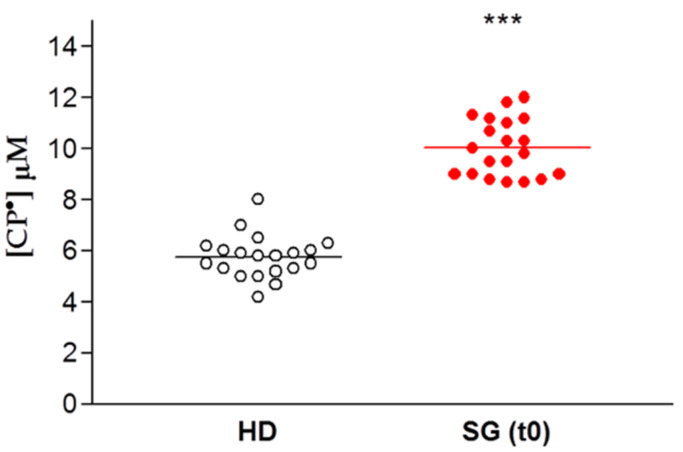
EPR- spin probing analysis of oxidative stress in blood. Healthy Donors (HD) vs. obese patient candidates for Sleeve Gastrectomy (SG). Lines indicate the mean value of CP• concentration, *** *p* < 0.01.

The data are expressed for each patient enrolled for our study as the concentration of CP• detected, both in controls (HD) and in obese patients prior to SG (SG) (Figure 3). There was a significant difference between these two groups, with increased formation of CP• in the SG group compared to the HD controls (9.9 ± 0.3 µM vs. 5.8 ± 0.2 µM respectively, *p* < 0.01), indicating an increased free radical concentration in the obese patients.

### 3.3. Identification of the Oxidative Species Responsible for Oxidative Stress in Obese Patients

**Figure 4:**
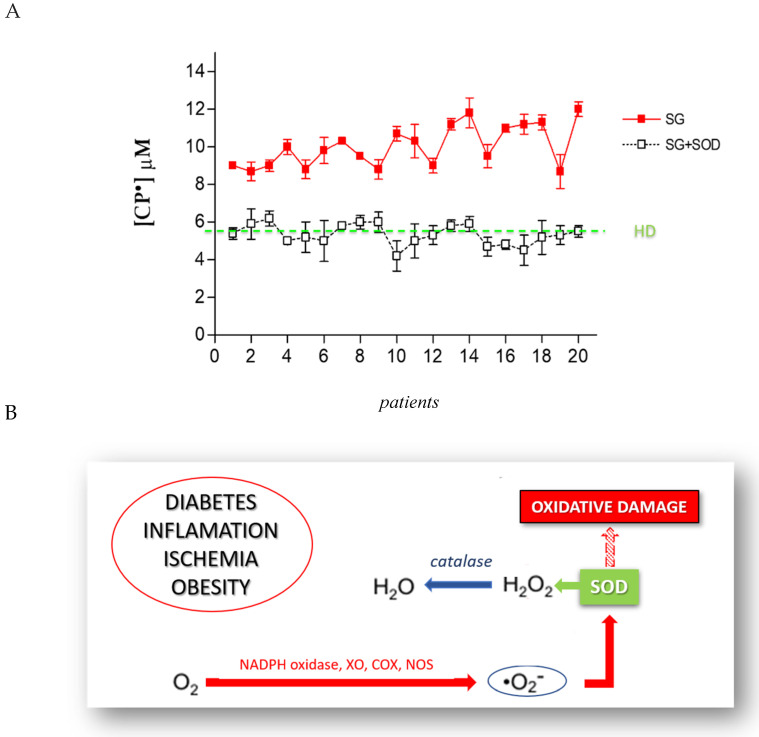
(**A**) EPR-spin probing analysis of the obese patients’ blood (SG), before (red squares) and after (white squares) the in vitro treatment with SOD. The green line represents the mean value of the CP• concentration detected in HD. (**B**) The mechanism through which SOD prevents the oxidative damage induced by O_2_^•^.

The formation of CP• is the direct consequence of an increase of pro-oxidant species. In order to identify the ROS involved in this increased oxidative stress, we used a series of antioxidant enzymes or inhibitor molecules, able to modulate the formation of the oxidant species. We incubated the blood of each patient candidate for SG with different antioxidant enzymes, performing an EPR-spin probing analysis before and after the treatment to detect any changes in the CP• concentration. The results obtained (Figure 4A) showed that the use of SOD (10 µg/mL), an enzyme able to counteract the oxidative effects of O_2_^•^, induced a significant decrease of the CP• concentration in all patients (*p* < 0.05). The values of the CP• concentration detected in the blood of SG patients (white squares) were comparable to those found in HD (green line). SOD catalyzes the dismutation of O_2_^•^ into ordinary molecular oxygen (O_2_) and hydrogen peroxide (H_2_O_2_), preventing the toxic effects due to the powerful pro-oxidant activity of this superoxide (Figure 4B). We tested other enzymes involved in counteracting oxidative stress, such as the suitable scavenger of H_2_O_2_ catalase (CAT; 10 µg/mL), the metal chelating agent diethylenetriamine pentaacetic acid (DTPA; 1 mM), and the inhibitor of nitric oxidesynthase N-monomethyl-L-arginine (NMA; 5 mM). These compounds did not significantly inhibit the CP• concentration.

### 3.4. Effect of SOD, Cat, DTPA, and PMA on CPH Oxidation

**Figure 5:**
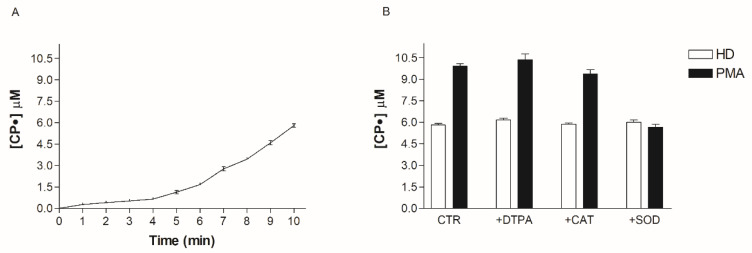
(**A**) Time-course of CP• radical formation. (**B**) Effect of DTPA (diethylenetriaminepentaacetic acid), CAT (catalase), SOD (superoxide dismutase) and PMA (Phorbol 12-Myristate 13-Acetate) on CPH (spin probe 1-hydroxy-3-carboxy-pyrrolidine) oxidation.

The above results using SOD suggest that O_2_^•^ is responsible for oxidative stress in obese patients. However, SOD could be paradoxically responsible for the increase of the CP• radical concentration in the blood, so demonstrating an increase of the CP• radical concentration may not reflect the increase in O_2_^•^ production. To better clarify the role of O_2_^•^ and the efficacy of our systems, we incubated the HD blood with all the enzymes and scavengers used to characterize the molecules involved in the oxidative stress pathways. The basal value of the CP• radical concentration (5.8 ± 0.2 µM) was not significantly modified by the addition of SOD, Cat, and DTPA, thereby excluding the direct involvement of oxidizing species and free metals in probe oxidation (Figure 5). To demonstrate the efficacy of our system to detect O_2_^•^ in the blood, we treated blood samples with PMA, a molecule able to stimulate WBC to produce O_2_^•^. The addition of PMA to the blood led to a rapid increase in CPH oxidation inhibited by SOD, suggesting that EPR-spin probing analysis is a reliable technique to detect the main oxidant species responsible for the oxidation of CPH in the blood. Cat and DTPA did not change the oxidation of CPH (Figure 5).

### 3.5. Effect of Sleeve Gastrectomy on Oxidative Stress

**Figure 6:**
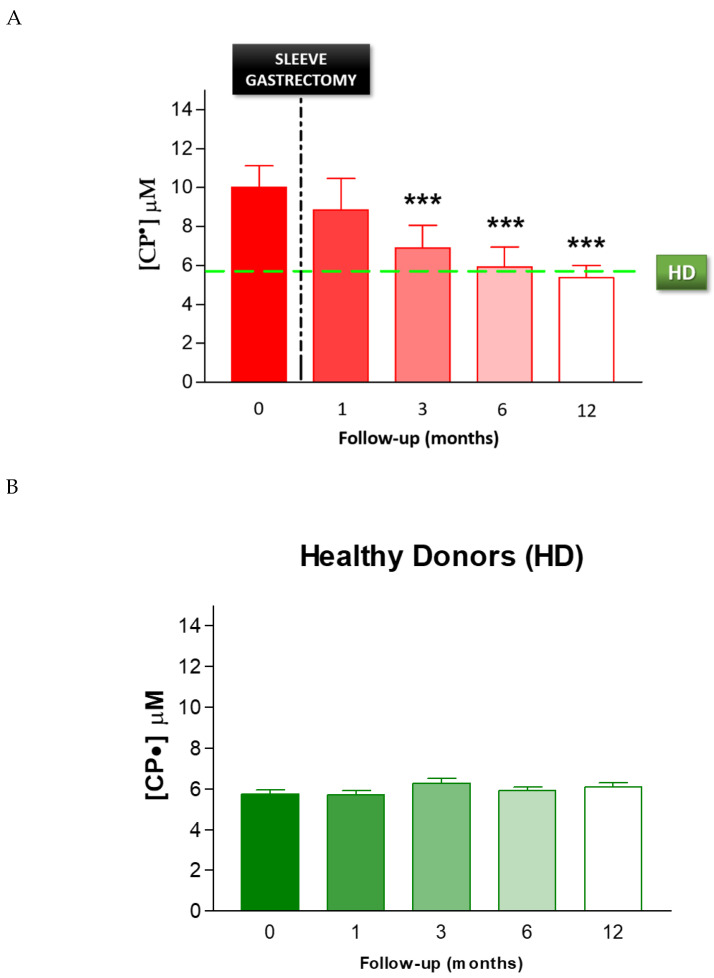
(**A**) EPR-spin probing analysis of oxidative stress in patients’ blood before (0) and after SG. The green line represents the mean value of the CP• concentration detected in the control group HD, *** *p* < 0.01. (**B**) EPR- spin probing analysis of oxidative stress in the control population (HD), no statistical difference in the CP• concentration was detected in HD during the follow-up.

To evaluate the effect of SG on oxidative stress, we performed serial analyses on patients’ blood following SG. The EPR-spin probing analysis showed a progressive decrease in the CP• concentration after SG (Figure 6A). In particular, we observed a significant decrease from pre-operative levels by 3 months after SG (*p* < 0.01). The EPR-spin probing analysis performed 6 and 12 months after SG continued to be significantly different compared to the values detected before surgery. The CP• concentrations at 6 months (6.0 ± 0.4 µM) and 12 months (5.4 ± 0.2 µM) were both similar to those detected in the control group, HD (5.8 ± 0.2 µM, green line), *p* < 0.01. No statistical difference in the CP• concentration was detected in HD during the follow-up (Figure 6B). Moreover, we performed a statistical analysis to verify a correlation between the weight and the blood concentration of CP• detected in patients, respectively, before and 1, 3, 6, and 12 months after sleeve gastrectomy. The statistical analysis showed a significative positive correlation *p* = 0.0013; C.I.: 0.8406 to 0.9993 (see Figure S1).

## 4. Discussion

In this study, we examined ROS production in blood samples of obese patients before and after SG, using the EPR spin probing technique. EPR spectroscopy directly detects and quantifies free radicals in blood samples, compared to other techniques that use only indirect measurements (i.e., conjugated dienes, hydroperoxides, and aldehydes) to evaluate oxidative injury. The use of EPR spectroscopy offers several advantages with respect to other methods: First, the opportunity to directly detect free radicals’ formation, as well as the relative simplicity of sample preparation and a short measurement time. In brief, this technique is based on the interaction between an external magnetic field and the magnetic moments of unpaired electrons of molecules present in the sample (free radicals). This interaction can cause a split of the electron energy levels, producing a signal directly proportional to the free radical concentration in the sample. The EPR signal generated by this splitting during the sample analyses is equal to the energy difference (ΔE) between the energy levels, which is described by the resonance condition: *ΔE = hν = gμBB0* (where *hν* is quantum of the electromagnetic wave energy, *g* is the value of the radical, *μB* is the Bohr magneton, and *B0* is the magnetic induction of an external magnetic field) [30]. EPR spectroscopy detects oxidizing species produced in tissues in both physiological and pathological conditions, and is extremely sensitive to changes in the redox state.

Here, we demonstrated that, compared to healthy controls, blood samples from obese patients prior to SG produced higher concentrations of ROS, particularly O_2_^•^. This result was supported by (i) the decreased intensity of CP• measured when SOD was added to patient blood samples, and (ii) by the absence of change measured in the presence of CAT, DTPA, and NMA, suggesting that H_2_O_2_, metal-catalyzed reactions and nitrogen-derived species, respectively, were not involved in the pro-oxidant status of these samples. The cyclic hydroxylamine CPH is a partially cell-permeable spin probe providing both extracellular and intracellular ROS monitoring, and it is not specific to a singular oxidant, but it is rather able to screen the totality of ROS produced in biological samples [31]. However, the ability of CPH to capture an SOD-inhibitable signal when blood cells were activated by PMA demonstrated that the spin probe is able to specifically identify O_2_^•^ when this is produced in a biological sample, and that this radical was effectively produced in the blood of obese patients.

Our findings support the known clinical association between obesity and a condition of chronic inflammation associated with an increased production of ROS [17]. Some studies report that the association between obesity and oxidative stress could be due to the role played by peroxisome proliferator-activated receptor-*γ* coactivator (PGC-1*α*), described as a regulator of some mitochondrial functions, such as ROS detoxification [32]. Korda et al. [33] described that leptin, an adipocyte-derived hormone, is elevated in obesity and can induce oxidative stress, playing a pivotal role in mediating a proinflammatory state in obesity [34]. An increase in ROS production may also contribute to insulin resistance. The augmentation of ROS has been shown to activate c-jun N terminal kinase 1 (JNK1) [35], which is implicated in obesity-induced insulin resistance and decreased compensatory insulin secretion, both of which are key features of type 2 diabetes [36]. Rodent models have also demonstrated an association between insulin resistance and an inflammatory state. In a rodent model of insulin resistance caused by obesity, the genetic ablation of TNFα, or its receptor, improved insulin sensitivity [37]. A mechanism by which TNF can induce the production of ROS is through the activation of the enzyme cyclo-oxygenase (COX-2) in activated macrophages mediated by its receptor Tumor necrosis factor receptor 1 (TNFR1) [38,39]. The redox-inflammatory processes linked to obesity can contribute to liver dysfunction, favoring the maintenance of high blood levels of triglycerides and cholesterol, frequently present in obese people [40].

Importantly, we found that when patients underwent SG, the formation of O_2_^•^ was significantly decreased in a time-dependent manner, so that at one-year follow-up after SG, the radical level was comparable to that of HD. Our findings highlight that the improvement in the redox state was detected after the significant reduction of BMI induced by SG, confirming the link between oxidative stress and obesity. Limitations of this pilot study include its small sample size, and the results will need to be confirmed in a larger cohort. Additionally, our cohort was predominately female, and significant differences have been found both in oxidative stress and antioxidant enzyme activity levels in males and females, which may have played a role in our study [41]. However, by age and sex matching our experimental and control cohort, we attempted to decrease potential confounding by these factors. Finally, other studies are needed to complete the identification of the catalogue of molecules involved in oxidative stress and obesity, as well as those that may be specifically affected by SG, or other bariatric surgical procedures.

## 5. Conclusions

Many obesity-related conditions, such as cardiovascular disease, insulin resistance, and cancer, have been associated with increased inflammation. Our study demonstrated direct evidence of increased free radical production in the blood of obese patients compared to healthy controls, which subsequently normalized following SG. Further analysis identified O_2_^•^ as the specific molecule likely responsible for this increased oxidative stress. Our findings demonstrate an additional benefit of bariatric surgery, and may provide a biochemical link to the many clinical associations demonstrated between obesity and inflammation-related disorders.

## Figures and Tables

**Table 1:** Sleeve gastrectomy outcomes and follow-up.

					Before	SG		1	M	After	SG	3	M	After	SG	6	M	After	SG	12	M	After	SG
**ID**	**AGE**	**SEX**	**H (m)**	**W (Kg)**	**BMI**	**AHT**	**OSA**	**W (Kg)**	**BMI**	**AHT**	**OSA**	**W (Kg)**	**BMI**	**AHT**	**OSA**	**W (Kg)**	**BMI**	**AHT**	**OSA**	**W (Kg)**	**BMI**	**AHT**	**OSA**
1	49	F	1.6	*106*	*41.4*	*n*	*0*	*89*	*34.8*	*n*	*0*	*72.8*	*28.4*	*n*	*0*	*65*	*25.4*	*n*	*0*	*52*	*20.3*	*n*	*0*
2	49	F	1.75	*140*	*45.7*	*n*	*1*	*113*	*36.9*	*n*	*1*	*95*	*31.0*	*n*	*0*	*85*	*27.8*	*n*	*1*	*82*	*26.8*	*n*	*0*
3	40	M	1.58	*103*	*41.3*	*n*	*0*	*91*	*36.5*	*n*	*0*	*71*	*28.4*	*n*	*0*	*68*	*27.2*	*n*	*0*	*63*	*25.2*	*n*	*0*
4	27	F	1.6	*110*	*43.0*	*n*	*0*	*95*	*37.1*	*n*	*0*	*75*	*29.3*	*n*	*0*	*71*	*27.7*	*n*	*0*	*65*	*25.4*	*n*	*0*
5	42	F	1.54	*107*	*45.1*	*n*	*0*	*97*	*40.9*	*n*	*0*	*78*	*32.9*	*n*	*0*	*70*	*29.5*	*n*	*0*	*60*	*25.3*	*n*	*0*
6	51	F	1.52	*108*	*46.7*	*n*	*0*	*95*	*41.1*	*n*	*0*	*72*	*31.2*	*n*	*0*	*71*	*30.5*	*n*	*0*	*67*	*29.0*	*n*	*0*
7	49	F	1.78	*120*	*37.9*	*y*	*2*	*103*	*32.5*	*y*	*2*	*80*	*25.2*	*y*	*1*	*77*	*24.3*	*y*	*0*	*76*	*24.0*	*y*	*0*
8	55	M	1.6	*93*	*36.3*	*y*	*0*	*85*	*33.2*	*y*	*0*	*74*	*28.9*	*y*	*0*	*64*	*25.0*	*n*	*0*	*60*	*23.4*	*n*	*0*
9	39	F	1.5	*91*	*40.4*	*n*	*0*	*82*	*36.4*	*n*	*0*	*75*	*33.3*	*n*	*0*	*66*	*29.3*	*n*	*0*	*58*	*25.8*	*n*	*0*
10	39	F	1.56	*105*	*43.1*	*n*	*0*	*96*	*39.3*	*n*	*0*	*81.5*	*33.5*	*n*	*0*	*74*	*30.4*	*n*	*0*	*67*	*27.5*	*n*	*0*
11	37	F	1.77	*144*	*46.0*	*n*	*0*	*123*	*39.3*	*n*	*0*	*98*	*31.3*	*n*	*0*	*88*	*28.1*	*n*	*0*	*85*	*27.1*	*n*	*0*
12	39	M	1.7	*135*	*46.7*	*n*	*0*	*115*	*39.8*	*n*	*0*	*98*	*33.9*	*n*	*0*	*85*	*29.4*	*n*	*0*	*78*	*27.0*	*n*	*0*
13	46	F	1.7	*110*	*38.1*	*y*	*0*	*103*	*35.6*	*y*	*0*	*88*	*30.4*	*y*	*0*	*78*	*27.0*	*y*	*0*	*72*	*24.9*	*n*	*0*
14	42	F	1.66	*125*	*45.4*	*n*	*2*	*114*	*41.4*	*n*	*2*	*97*	*35.2*	*n*	*2*	*85*	*30.8*	*n*	*1*	*76*	*27.6*	*n*	*0*
15	42	M	1.83	*129*	*38.5*	*y*	*0*	*115*	*34.3*	*y*	*0*	*102*	*30.5*	*y*	*0*	*88*	*26.3*	*y*	*0*	*72*	*21.5*	*n*	*0*
16	42	F	1.65	*115*	*42.2*	*n*	*0*	*98*	*36.0*	*n*	*0*	*82*	*30.1*	*n*	*0*	*71*	*26.1*	*n*	*0*	*65*	*23.9*	*n*	*0*
17	51	F	1.62	*106*	*40.4*	*n*	*0*	*88*	*33.5*	*n*	*0*	*71*	*27.1*	*n*	*0*	*65*	*24.8*	*n*	*0*	*59*	*22.5*	*n*	*0*
18	39	F	1.5	*98*	*43.6*	*n*	*0*	*89*	*39.6*	*n*	*0*	*79*	*35.1*	*n*	*0*	*68*	*30.2*	*n*	*0*	*57*	*25.3*	*n*	*0*
19	49	F	1.7	*123*	*42.6*	*n*	*0*	*105*	*36.3*	*n*	*0*	*88*	*30.4*	*n*	*0*	*76*	*26.3*	*n*	*0*	*65*	*22.5*	*n*	*0*
20	40	F	1.68	*118*	*41.8*	*n*	*0*	*91*	*32.2*	*n*	*0*	*86*	*30.5*	*n*	*0*	*76*	*26.9*	*n*	*0*	*72*	*25.5*	*n*	*0*
**Mean**	**43.4**			**114.3**	**42.3**			**99.3**	**36.8**			**83.2**	**30.8**			**74.5**	**27.7**			**67.6**	**25.0**		
*±SD*	*6.5*			*14.8*	*3.1*			*11.6*	*2.9*			*10.2*	*2.6*			*8.0*	*2.1*			*8.9*	*2.2*		
*min*	*27.0*			*91.0*	*36.3*			*82.0*	*32.2*			*71.0*	*25.2*			*64.0*	*24.3*			*52.0*	*20.3*		
*max*	*55.0*			*144.0*	*46.7*			*123.0*	*41.4*			*102.0*	*35.2*			*88.0*	*30.8*			*85.0*	*29.0*		

The results express the clinical evaluations before and 1, 3, 6, and 12 months (M) after Sleeve Gastrectomy (SG). Height (H), Weight (W), Body Mass Index (BMI), Obstructive Sleep Apnea (OSA, 0 = absence, 1 = mild, 2 = moderate, 3 = severe, according The Report of an American Academy of Sleep Medicine Task Force [28]), while yes/no (y/n) indicates the assumption of AHT (Anti-Hypertensive Therapy).

## Supplementary Materials

Supplementary materials can be found at https://www.mdpi.com/2227-9059/8/6/168/s1.
