# Supplementary material for: The Effect of Sleeve Gastrectomy on Oxidative Stress in Obesity
Source: Biomedicines. 2020 Jun 19;8(6):168. doi: 10.3390/biomedicines8060168 (PMC7344505; doi:10.3390/biomedicines8060168)
Supplement: Supplementary file 1 [file biomedicines-08-00168-s001.pdf]

Correlation between weight and the blood concentration of CP-

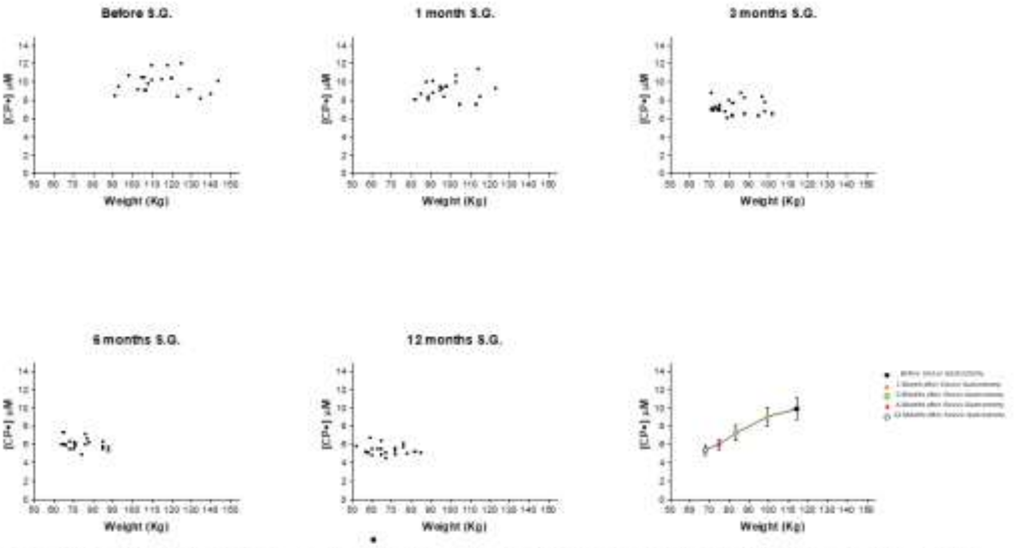

The correlation analyses Weight/CP- at each time point following sleeve gastrectomy (S.G) showed no statistically significant results for each time-point, while the correlation Weight/CP- resulted statistically significant (correlation p=0.0013; C.I. 0.8406 to 0.9995), considering the time-course of the mean values of Weight/CP- during the follow-up (last figure).
